# Characterization of the complete chloroplast genome and comparative analysis of the phylogeny and codon usage bias of three Yunnan wild rice species

**DOI:** 10.3389/fpls.2025.1555104

**Published:** 2025-07-02

**Authors:** Rongxin Li, Bo Wang, Suqin Xiao, Ling Chen, Fuyou Yin, Jinlu Li, Cong Jiang, Dunyu Zhang, Qiaofang Zhong, Yun Zhang, Jiaxin Xing, Zaiquan Cheng, Li Liu

**Affiliations:** ^1^ Biotechnology and Germplasm Resources Institute, Yunnan Academy of Agricultural Sciences, Kunming, China; ^2^ School of Agriculture, Yunnan University, Kunming, China; ^3^ Yunnan Provincial Key Lab of Agricultural Biotechnology, Yunnan Academy of Agricultural Sciences, Kunming, China

**Keywords:** Yunnan wild rice species, chloroplast genome, phylogeny, codon usage bias, mutation pressure, natural selection

## Abstract

**Background:**

Wild rice species with a rich genetic diversity, wide adaptability, and high photosynthetic rates provide a valuable genetic reservoir for rice breeding.

**Methods:**

In this study, the chloroplast (cp) genomes of three Yunnan wild rice species, namely *Oryza rufipogon*, *Oryza officinalis*, and *Oryza granulata*, were sequenced using second-generation sequencing technology, followed by assembly and annotation. Phylogeny and codon usage bias were analyzed using MEGA and RStudio.

**Results:**

The total lengths of the cp genomes of the three species ranged from 134,556 to 135,937 bp, with a GC content of 39.0%. The large single-copy region of *Oryza granulata* was 2000 bp longer than that of the other two species. In total, 133 genes were identified in the cp genome, including domestication genes *psbZ*, *ycf68*, and *lhba*. Phylogenetic analysis showed that *Oryza rufipogon* is distinct from the Indian *Oryza nivara*, *Oryza officinalis* evolved from *Oryza australiensis*, and *Oryza granulata* shared a closer relationship with *Oryza brachyantha*. The average effective number of codons of the three species was above 45, indicating weak codon usage bias.

**Conclusion:**

Analysis of the ENC, PR2, and neutrality plots revealed that natural selection played a significant role in the chloroplast codon usage bias of the three species, mainly regulating genes involved in self-replication and photosynthesis. Fourteen optimal codons were identified, with 13 ending in A/U and one ending in C. These results are crucial for mining favorable genes related to photosynthesis and investigating the evolution of wild rice species. Overall, this study provides valuable information on the genomic composition and genetics of three Yunnan wild rice species.

## 1 Introduction

Chloroplasts (cps) are the main photosynthetic organelles in plants that provide the materials and energy needed to sustain life (Kirchhoff, 2019). They contain a stable and relatively conserved genome, independent of the nuclear genome, with distinctive characteristics, including complex patterns of mutational changes in non-coding regions, rich genetic variation, and a conservative evolution rate. Thus, cp genomes are ideal for molecular evolution studies, species phylogenetic relationships, and gene identification. Recently, cp genomic data have significantly expanded owing to the availability of high-throughput sequencing technology with a lower cost of sequencing. The cp genomes of *Oryza* (Gao et al., 2019), *Cinnamomum* (Bandaranayake et al., 2023), *Cynanchum acutum* (Zhang et al., 2023), *Cichorium endivia* L (Testone et al., 2023). and *Pedicularis* (Wang et al., 2024a) have been sequenced to reveal genetic relationships within and between species at the molecular level, aiding the verification of traditional evolutionary theory and classification and reclassification of some controversial species.

Wild rice, comprising 22 species worldwide, harbors valuable genetic resources for modern rice breeding (Gao et al., 2019). Three wild rice species, *Oryza rufipogon*, *Oryza officinalis*, and *Oryza granulata*, have been found and collected from various sites in southern China, such as Yunnan Province (Zheng et al., 2024). Owing to their adaptation to harsh environmental conditions for prolonged natural selection under stress and preservation of many desirable and original traits, wild rice possesses numerous beneficial genes such as those related to disease, insect, and pest resistance; tolerance to shade; high photosynthetic efficiency; and biomass formation (Chen et al., 2022; Lu et al., 2022; Lei et al., 2023; Kan et al., 2024; Xie et al., 2024). Previously, we compared the photosynthetic efficiency of the three wild rice species and *Oryza sativa* and found that *Oryza officinalis* exhibited over 20 times higher biomass accumulation than *Oryza sativa*, and its carboxylation efficiency and light-attenuation point were more than twice that of cultivated rice (Li et al., 2021). *Oryza granulata* is the only upland wild rice species that grows in bamboo thickets, mixed wood forests of shrubs and trees, damp locations beside streams, waterfalls, or seasonally dry, sloping lands with complete shade (Chen et al., 2024). Therefore, shaded plants exhibit photosynthetic characteristics. Very few genes related to the photosynthetic pathways have been identified in wild rice (Li et al., 2021; Zheng et al., 2024). The cp genome of 22 *Oryza* species have been sequenced to reveal to understand the diversity, evolution, and adaptation to diverse ecological habitats from the basic difference of genetic structure (Gao et al., 2019). However, the cp genome evolution of the three Yunnan wild rice species with some *Oryza* species such as *Oryza schlechteri*, *Oryza coarctata* and Dongxiang wild rice remains largely unknown, and knowledge of the genetic diversity of genes involved in the photosynthetic pathway among wild rice species and cultivated rice remains limited.

Genetic codons, a set of rules defining DNA translated into amino acid sequences, carry information to determine the genomic stability and genetic variation of a species. During transcription and translation, the same amino acid may be encoded by different codons known as synonymous codons. With respect to gene expression, synonymous codons for the same amino acid are not used with equal frequencies in genomes; this is termed as codon usage bias (Chi et al., 2020). Codon usage bias is manifested in the genome evolution of organisms adapting to different environments. It has evolved through mutations, natural selection, and genetic drift in various organisms (Hao et al., 2022) and is vital for gene expression by regulating transcription and translation (Parvathy et al., 2022). Optimal codons regulate the rhythm of elongation to facilitate co-translational polypeptide folding, significantly impacting differential protein production and folding, and are associated with increased synthesis efficiency of polypeptide chains (Pechmann and Frydman, 2013). Therefore, investigating codon usage bias in cp genomes is beneficial for revealing species evolution and genetic relationships and improving gene expression efficiency in genetic transformation research. Codon usage bias has been widely used in the study of various organisms of *Annonaceae*, *Theaceae*, ciliates, cherries, citrus, *Aconitum* species, and *Epimedium* species (Hu et al., 2024). However, little is known about its role in the cp genomes of wild rice species.

This study aimed to sequence, assemble into scaffolds, and annotate the cp genomes of three Yunnan wild rice species to extract candidate genes and analyze codon usage bias factors. Factors influencing codon usage bias, codon optimality, and phylogenetic relationships were analyzed using CodonW, RStudio, and MEGA. Overall, this study aimed to elucidate the genetic basis of the Yunnan wild rice species, offer crucial insights for understanding the evolution and genetic relationships of wild rice species. Furthermore, this study aimed to provide breeders valuable genetic stocks for constructing effective vectors of favorable genes coding Rubisco within chloroplasts such as CcmM35 and Se7942 genes to increase yield, and coding Trehalose 6-phosphate (T6P) synthase and Trehalose phosphate synthase (TPS1) to enhance drought resistance, modifying nuclear-encoded chloroplast-targeted genes (NECG) expression, obstructing protein delivery to the chloroplast, or inside the chloroplast to straightaway disrupting its function to improve disease resistance in cultivated rice.

## 2 Materials and methods

### 2.1 Plant materials and DNA extraction

Three Yunnan wild rice species, *Oryza rufipogon*, *Oryza officinalis*, and *Oryza granulata*, preserved in the Kunming National Wild Rice Germplasm Resources Nursery of China. A previously reported modified high salt method was utilized to extract cpDNA (Shi et al., 2012; Gao et al., 2019). 100 g (fresh weight) leaves of each wild rice species were collected and homogenized with Buffer A (1.25 M NaCl, 0.25 M ascorbic acid, 10 mM sodium metabisulfite, 0.0125 M Borax, 50 mM Tris-HCl (pH 8.0), 7 mM EDTA, 1% PVP-40 (w/v), 0.1% BSA (w/v), 1 mM DTT) for 30 seconds. The homogenate was filtered into centrifuge bottles using two layers of Miracloth (Merck) by softly squeezing the cloth and then centrifuged at 200 g for 20 min to descard cell debris. The supernatant was centrifuged at 3500 g for 20 min. The resulting chloroplast pellet was washed with Buffer B (1.25 M NaCl, 0.0125 M Borax, 1% PVP-40 (w/v), 50 mM Tris-HCl (PH 8.0), 25 mM EDTA, 0.1% BSA (w/v), 1 mM DTT) and followed centrifugation at 3750 g for 20 min to get pure chloroplasts. Then the chloroplasts were lysed using Buffers C (100 mM NaCl, 100 mM Tris-HCl (PH 8.0), 50 mM EDTA, 1 mM DTT) with SDS and Proteinase K at 55°C for at least 4 hours and centrifuged to descard pellet. Treated with 5 M KAc, the supernatant was extracted cpDNA by Phenol and Chloroforml-lsoamyl-Alcohol and precipitated with Isopropyl alcohol. Finally, the cpDNA was resuspended with TE buffer. To make sure the genomic cpDNA satisfied the criteria for sequencing, their quality and concentration were assessed using a microspectrophotometer (Nanodrop 2000, USA), and total DNA quality was detected via 1% agarose gel electrophoresis.

### 2.2 Sequencing, cp genome assembly, and annotation of Yunnan wild rice species

The qualified DNA samples were broken into fragments of about 500 bp by ultrasonic DNA fragmentation, and then the fragments were purified and end-repaired to construct a chloroplast genome library. The Raw data was obtained by sequencing on the Illumina Hiseq TM2000 platform following the manufacturer’s protocol, with a reading length of 150 bp. Quality control of the raw sequencing data was performed using Fastp v0.23.4 (https://github.com/OpenGene/fastp) with parameters of -Q -y -G -Y 10 –adapter_fasta adapter.fa -l 60 -b 150 (Chen, 2023). The clean data were assembled in the cp genome using GetOrganelle v1.7.7.1 (https://github.com/Kinggerm/GetOrganelle) with k-mer parameters of 21,77 and 127 (Jin et al., 2020). After alignment and correction, the complete cp genome sequences of three Yunnan wild rice species were obtained. The cp genome sequence of *Oryza sativa* L. ssp. *Nipponbare* (MW001303.1) was downloaded from the National Center for Biotechnology Information database (https://www.ncbi.nlm.nih.gov/). Next, the cp genome was annotated using CPGAVAS2 (http://47.96.249.172:16019/analyzer/annotate) with default parameters of MSR (1-10 2-6 3-5 4-5 5-5 6-5), TR (2 7 7 80 10 50 500 -f -d –m), DR (-f -p -h 3 -l 30), and taking 43-genome-model as reference dataset (Shi et al., 2019). Gene annotations were compared using BLAST, followed by manual adjustment using Geneious v9.0.2 software (Wu et al., 2025). The distribution of annotated genes among the cp genomes was calculated and visualized using Chloroplot (https://irscope.shinyapps.io/Chloroplot/) (Zheng et al., 2020). The data reported in this paper have been deposited in the GenBase in National Genomics Data Center, Beijing Institute of Genomics, Chinese Academy of Sciences/China National Center for Bioinformation, under accession number C_AA107112.1 to C_AA107114.1 that is publicly accessible at https://ngdc.cncb.ac.cn/genbase.

### 2.3 Phylogenetic analysis

A total of 25 cp genome sequences from the Yunnan wild rice species assembled in this study and other wild rice species from different regions, two cultivated rice species of Indica and Japonica, with *Arabidopsis thaliana* as the outgroup downloaded from NCBI, were used for phylogenetic analysis. First, the 25 cp genome sequences were subjected to collinearity analysis using Circoletto (https://bat.infspire.org/circoletto/) (Darzentas, 2010) to identify the quadripartite structure and remove reverse duplications or rearrangements. Then, the sequences were aligned using MAFFT v7.505 with the default parameter –auto of –localpair –maxiterate 1000 –retree 2–maxiterate 0 -op 1.53 -ep 0.0 -h -d 1 -p -n 5 -t 4 -o (Katoh and Standley, 2013; Waswa et al., 2023). The filtered sequences were imported into MEGA11 v11.0.13 (https://www.megasoftware.net/) to create a phylogenetic tree using the maximum likelihood method. The best-fit model was screened using the FIND BEST MODEL, and the repeat parameter was bootstrap-1000; that is, the cycle was loaded 1,000 times. The phylogenetic tree was trimmed using Adobe Illustrator 2023 and ITOL v6 (https://itol.embl.de/) (Letunic and Bork, 2021).

### 2.4 Codon usage bias analysis

#### 2.4.1 Extraction of coding sequence

The coding sequence (CDS) from the cp genomes of four species was extracted using Python script and selected to meet the following criteria: sequence length ≥ 300 bp, with an ATG start codon and a stop codon (TGA/TAG/TAA). The GC content, including GC1, GC2, and GC3, represents the counts of G and C nucleotides at the first, second, and third positions of each codon in the gene were calculated using CUSP (https://www.bioinformatics.nl/cgi-bin/emboss/cusp) (Shen et al., 2024), codon adaptation index (CAI) (Sharp and Li, 1987). The effective number of codons (ENC, observed value, ENCobs), relative synonymous codon usage (RSCU), and base frequency at the third codon position were analyzed by CodonW (v1.4.2). The correlation analysis was performed using RStudio.

#### 2.4.2 Analysis of factors influencing codon usage bias

Codon usage bias is commonly assessed using neutral, ENC- and PR2-plot analyses. Neutral plot analysis was used to examine the relationship between GC12, the mean GC content at the first and second positions of each codon in the gene, and GC3. The GC3 value was used as the abscissa, and GC12 was used as the ordinate to draw the scatter plot. ENC-plot analysis was used to investigate the influence of mutations or other factors on codon usage. An ENC-plot was generated with the ENC value (The expectations of ENC) on the ordinate and GC3 on the abscissa. The expected curve was calculated to assess the effects of mutation and codon usage bias using the formula: ENCexp = 2 + GC3 + 29/[GC3^2^ + (1-GC3)^2^] (Sueoka, 1995).

The PR2-plot analysis was used to explore the proportional relationship between purines and pyrimidines at the third base of each four-codon degenerate amino acid. The PR2-plot was drawn with A3/(A3 + T3) as the vertical axis and G3/(G3 + C3) as the horizontal axis, where A3, T3, G3, and C3 denote the content of A, T, G, and C in the third codon position, respectively. The preferred orientation and strength of the A = T and G = C biases at the third codon position were analyzed using vectors extending from the center point to other points (Zhang and Feng, 2025).

#### 2.4.3 Determination of optimal codons

The ENC values of the CDS of Yunnan wild rice species were sorted from low to high, and 10% of the genes were selected from both ends to construct high- and low-expression libraries, according to the method described by Zhang et al. (2021). The RSCU and ΔRSCU values were then calculated for each group, with Δ RSCU ≥ 0.08 (Δ RSCU = RSCU high-RSCU low) indicating high-expression codons, and codons with RSCU > 1 denoting high-frequency codons. Codons meeting both criteria were defined as optimal codons.

## 3 Results

### 3.1 The cp genomic features of Yunnan wild rice species

**Figure 1:**
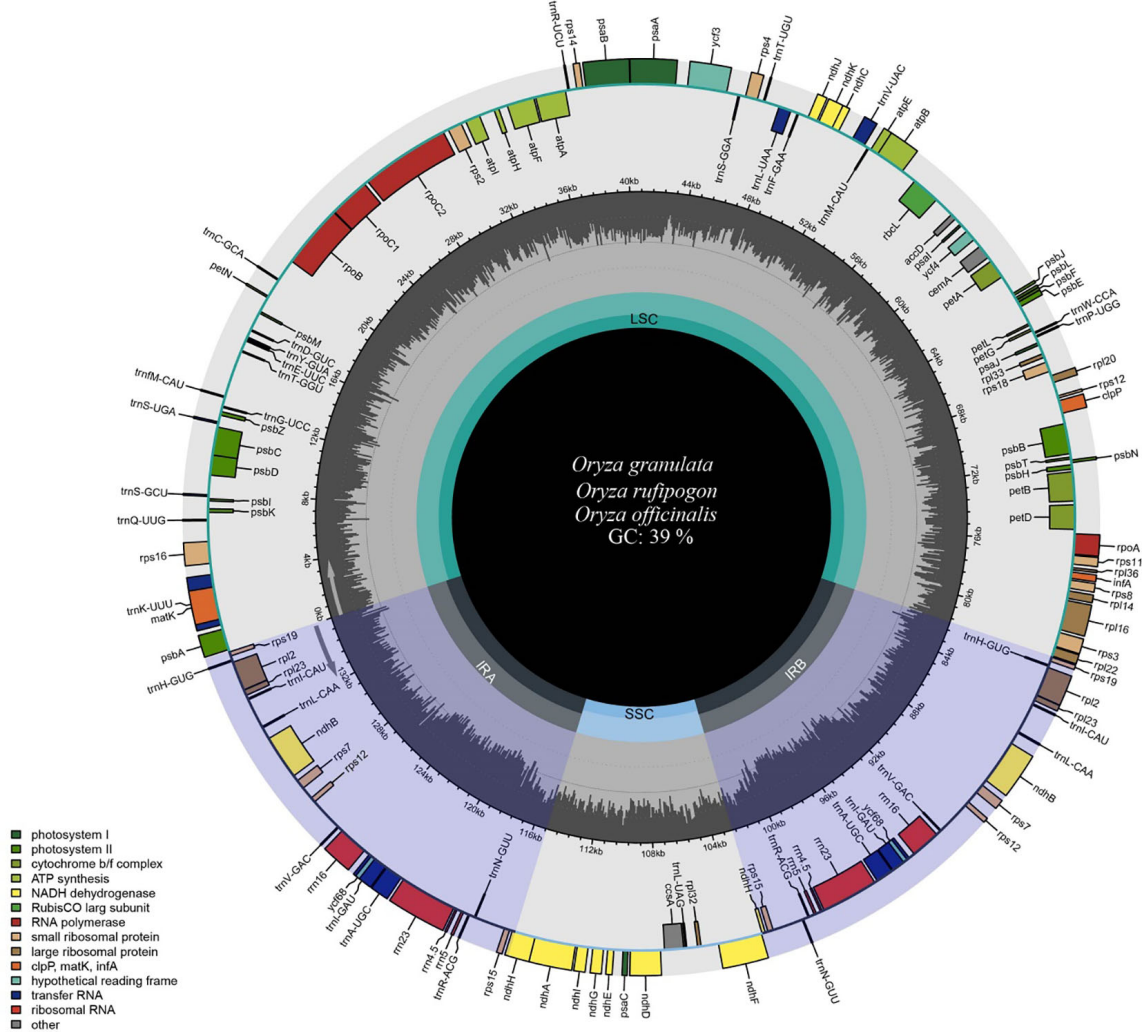
Map of chloroplast genomes of Yunnan wild rice species. Genes inside the circle show clockwise transcription, whereas those outside the circle show anti-clockwise transcription. Genes belonging to a different functional group are showed in different colors. The gray plots in the middle circle represent GC contents. The inner circle displays the reverse repeat regions as well as the small single-copy and large single-copy regions. The circular gene map was drawn using Chloroplot.

Filtering the raw sequencing data of *Oryza rufipogon*, *Oryza officinalis*, and *Oryza granulata* yielded high-quality clean data of 1.73 Gb, 1.29 Gb, and 1.8 Gb, respectively. The base quality percentages for Q30 were 91.22%, 92.67%, and 93.67%, showed as **Supplementary Table 1**. The whole cp genomes of the three species varied from 134,587 to 135,937 bp in length (**Table 1**), with an overall GC content of approximately 39.0% (**Figure 1**). Notably, the cp genome size of the Yunnan wild rice species was larger than that of the cultivated rice species with the size of 134,556 bp which confirmed previous finding that cp genome sizes of all 21 *Oryza* species was larger than L. *japonica* (cultivated rice), possibly responsible for the high photosynthetic efficiency or resistance to shade in wild rice species.

**Table 1:** Information regarding cp genome structures of four species.

Species	Total length/bp	LSC/bp	SSC/bp	IR/bp	LSC GC%	SSC GC%	IR GC%
*O. rufipogon*	134,587	80,636	12,347	20,802	37.11	33.35	44.35
*O. officinalis*	134,824	80,852	12,330	20,821	37.11	33.37	44.34
*O. granulata*	135,937	81,837	12,498	20,801	37.10	33.32	44.33
*O. sativa*	134,556	80,603	12,347	20,803	37.10	33.37	44.34

cp, chloroplast; LSC, large single-copy; SSC, small single-copy; IR, inverted repeat.

The cp genomes of the three species displayed a quadripartite structure comprising two inverted repeat regions (IRA and IRB) separated by large single-copy (LSC) and small single-copy (SSC) regions. The LSC region varied from 80,603 bp to 81,837 bp in length, whereas the SSC region ranged from 12,330 bp to 12,498 bp. The IR region ranged from 20,801 to 20,821 bp. Notably, the LSC and SSC lengths of *Oryza rufipogon*, with a closer relationship with the cultivated rice *Oryza sativa*, were similar to those of *Oryza sativa*. The GC content differed significantly across the three regions (LSC at 37%, SSC at 33%, and IR at 44%), aligning with typical angiosperm GC content.

The cp genomes of all three species were annotated. A total of 133 genes, including 8 rRNA, 37 tRNA, and 89 CDS genes, were identified (**Table 2**). Among the 133 genes, 15 contained introns, including five tRNA genes *(trnA-UGC*, *trnI-GAU*, *trnK-UUU*, *trnL-UAA*, and trnV-UAC) and ten protein-coding genes *(atpF*, *petB*, *petD*, *ndhA*, *ndhB*, *rpl2*, *rpl16*, *ycf3*, *rps12* and *rps16)*. *ycf3* had two introns, whereas the other 14 genes had only one intron. Gene duplications were observed in 22 protein-coding genes. As for the function, the cp genes were involved in photosynthesis, self-replication, other functions, and unknown functions. The cp genes, namely *psbZ*, *petL*, *ycf68*, and *lhba*, were highly conserved among the four species. A comparison of the cp genes between wild and cultivated rice species revealed that *psbZ*, *ycf68*, and *lhba* may be related to the domestication of rice species.

**Table 2:** Gene annotation of cp genomes of four species.

Category	Gene group	Gene symbol	*O. rufipogon*	*O. officinalis*	*O. granulata*	*O. sativa*
Photosynthesis	Subunits of photosystem I	*psaA*, *psaB*, *psaC*, *psaI*, *psaJ*	S	S	S	S
	Subunits of photosystem II	*psbA*, *psbB*, *psbC*, *psbD*, *psbE*, *psbF*, *psbH*, *psbI*, *psbJ*, *psbK*, *psbL*, *psbM*, *psbN*, *psbT*, *psbZ*	S	S	S	-*psbZ*
	Subunits of NADH dehydrogenase	ndhA*, ndhB*(2), *ndhC*, *ndhD*, *ndhE*, *ndhF*, *ndhG*, *ndhH*(2), *ndhI*, *ndhJ*, *ndhK*	S	S	S	S
	Subunits of cytochrome b/f complex	*petA*, petB*, petD*, *petG*, *petL*, *petN*	*-petL*	*-petL*	S	S
	Subunits of ATP synthase	*atpA*, *atpB*, *atpE*, atpF*, *atpH*, *atpI*	S	S	S	S
	Large subunit of rubisco	*rbcL*	S	S	S	S
Self-replication	Large ribosomal subunit	*rpl14*, rpl16*, rpl2*(2), *rpl20*, *rpl22*, *rpl23*(2), *rpl32*, *rpl33*, *rpl36*	S	S	S	S
	Small ribosomal subunit	*rps11*, rps12*(3), *rps14*, *rps15*(2), rps16*, *rps18*, *rps19*(2), *rps2*, *rps3*, *rps4*, *rps7*(2), *rps8*	S	S	S	S
	Subunits of RNA polymerase	*rpoA*, *rpoB*, *rpoC1*, *rpoC2*	S	S	S	S
	Ribosomal RNAs	*rrn16S*(2), *rrn23S*(2), *rrn4.5S*(2), *rrn5S*(2)	S	S	S	S
	Transfer RNAs	trnA-UGC*(2), *trnC-GCA*, *trnD-GUC*, *trnE-UUC*, *trnF-GAA*, *trnG-UCC*, *trnH-GUG*(2), *trnI-CAU*(2), trnI-GAU*(2), trnK-UUU*, *trnL-CAA*(2), trnL-UAA*, *trnL-UAG*, *trnM-CAU*, *trnN-GUU*(2), *trnP-UGG*, *trnQ-UUG*, *trnR-ACG*(2), *trnR-UCU*, *trnS-GCU*, *trnS-GGA*, *trnS-UGA*, *trnT-GGU*, *trnT-UGU*, *trnV-GAC*(2), trnV-UAC*, *trnW-CCA*, *trnY-GUA*, *trnfM-CAU*	S	S	S	S
Other gene	Maturase	*matK*	S	S	S	S
	Protease	*clpP*	S	S	S	S
	Envelope membrane protein	*cemA*	S	S	S	S
	Acetyl-CoA carboxylase	*accD*	S	S	S	S
	Synthesis factor of c-cytochrome	*ccsA*	S	S	S	S
	Initiation factor of translation	*infA*	S	S	S	S
Unknown function	Conserved open reading frame	ycf3**, *ycf4*, *ycf68*(2), *lhba*	*-lhba*	*-lhba*	*-lhba*	*-ycf68*

*, one intron; **, two introns; (2), number of copies of multi-copy genes; -, non-gene; S, same gene.

### 3.2 Phylogenetic analysis of the cp genome of Yunnan wild rice species

**Figure 2:**
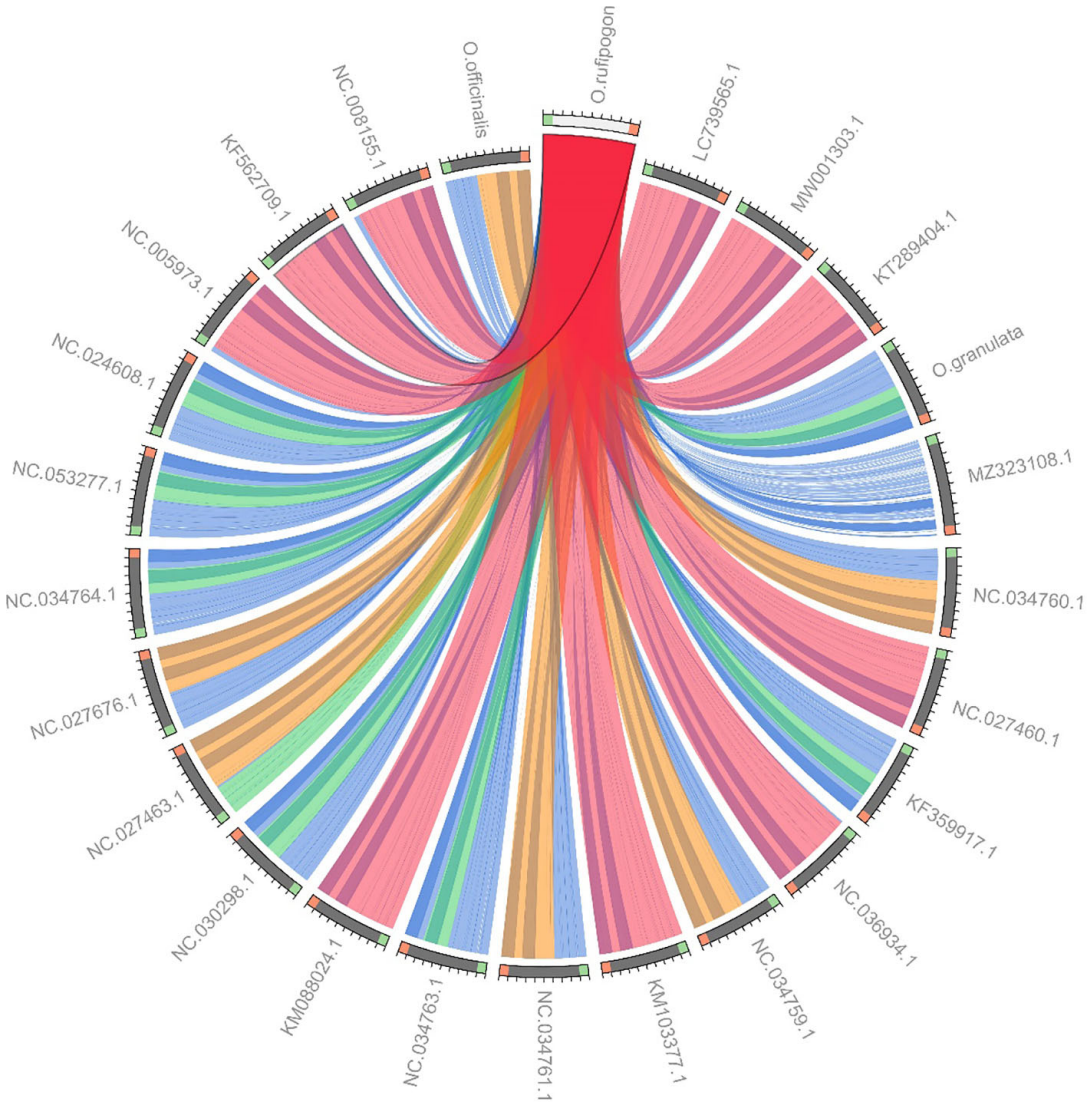
Collinearity analysis of chloroplast genomes in 24 *Oryza* species and *Arabidopsis thaliana*. LC739565.1, *Oryza sativa* Japonica Group; MW001303.1, *Oryza sativa* temperate japonica; KT289404.1, *Oryza sativa* tropical japonica; MZ323108.1, *Arabidopsis thaliana*; NC034760.1, *Oryza alta*; NC027460.1, *Oryza barthii*; KF359917.1, *Oryza brachyantha*; NC036934.1, *Oryza coarctata*; NC034759.1, *Oryza eichingeri*; KM103377.1, *Oryza glaberrima*; NC034761.1, *Oryza grandiglumis*; NC034763.1, *Oryza longiglumis*; KM088024.1, *Oryza longistaminata*; NC030298.1, *Oryza minuta*; NC027463.1, *Oryza officinalis*; NC027676.1, *Oryza punctate*; NC034764.1, *Oryza ridleyi*; NC053277.1, *Oryza schlechteri*; NC024608.1, *Oryza australiensis*; NC005973.1, *Oryza nivara*; KF562709.1, *Oryza rufipogon* (Dongxiang); NC008155.1, *Oryza sativa* Indica Group. Collinearity blocks with minimum length of 1 kb were used for the collinearity analysis. *O. rufipogon*, *O. officinalis*, *Oryza sativa* Japonica Group (LC739565.1) had good linearity. *O. officinalis* and *Oryza sativa* Indica Group (NC008155.1), *O. granulata* and *Oryza sativa* tropical japonica (KT289404.1) had good linearity.

**Figure 3:**
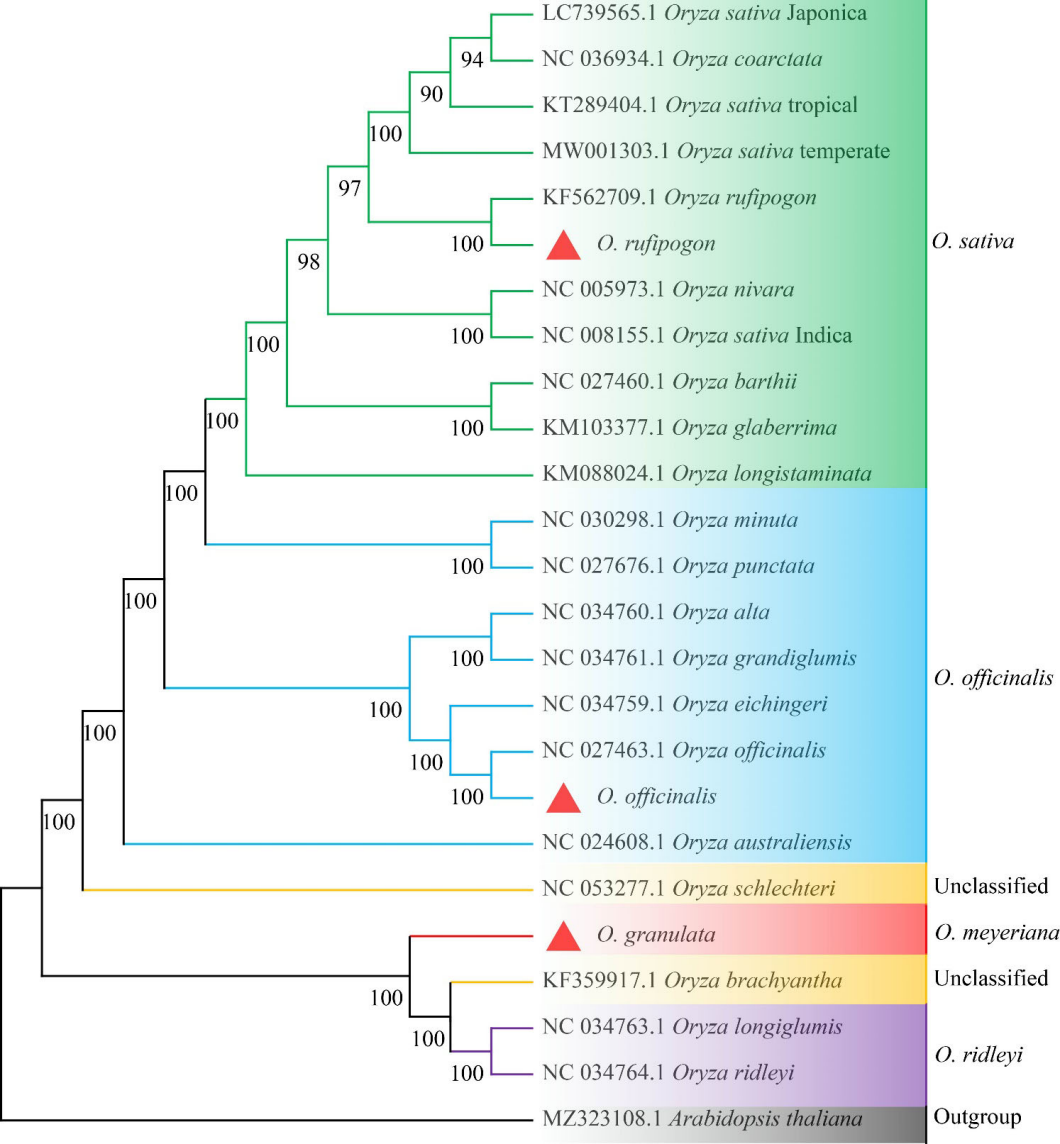
Whole-genome phylogenetic tree of chloroplasts of 24 *Oryza* species with *Arabidopsis thaliana* set as the outgroup. Maximum likelihood method was used to reconstruct the tree. The value of ML supports was shown above the branches. The cp genomes newly sequenced in this study are highlighted with red triangle marks.

Owing to a high number of cp genomes of wild rice species, such as *Oryza schlechteri*, *Oryza coarctata*, and *Oryza rufipogon* (Dongxiang) and cultivated rice species available, we reconstructed the phylogenetic tree of the wild rice species using MEGA software. A total of 25 cp genomes were subjected to phylogenetic analysis: the cp genome of the Yunnan wild rice species obtained in this study, 16 cp genomes of other wild rice species, five cp genomes of cultivated rice species, and the cp genomes of *Arabidopsis thaliana* obtained from NCBI (**Table 3**). The cp genomes were subjected to collinearity analysis, and the results showed no coding rearrangements or inversions in the cp genomes. Thus, they could be used to construct an accurate phylogenetic tree (**Figure 2**). Phylogenetic analysis showed that Yunnan *Oryza rufipogon* and Dongxiang *Oryza rufipogon* (KF562709.1) clustered into one branch, and Yunnan *Oryza officinalis* and *Oryza officinalis* (NC027463.1) downloaded from NCBI were within one clade with 100% bootstrap support (**Figure 3**). The consistency validated the quality of the assembled cp genome of Yunnan wild rice species, and proven that the cp genome in plants showed high level of conservation especially in one species.

**Table 3:** Species information for constructing the phylogenetic tree.

GenBank code	Species	GenBank code	Species
MZ323108.1	*Arabidopsis thaliana*	NC027676.1	*Oryza punctata*
NC034764.1	*Oryza ridleyi*	NC030298.1	*Oryza minuta*
NC034763.1	*Oryza longiglumis*	KM088024.1	*Oryza longistaminata*
KF359917.1	*Oryza brachyantha*	KM103377.1	*Oryza glaberrima*
NC053277.1	*Oryza schlechteri*	NC027460.1	*Oryza barthii*
NC024608.1	*Oryza australiensis*	NC005973.1	*Oryza nivara*
NC027463.1	*Oryza officinalis*	KF562709.1	*Oryza rufipogon* (Dongxiang)
NC034759.1	*Oryza eichingeri*	NC008155.1	*Oryza sativa* Indica Group
NC034761.1	*Oryza grandiglumis*	MW001303.1	*Oryza sativa* temperate japonica
NC034760.1	*Oryza alta*	KT289404.1	*Oryza sativa* tropical japonica
NC036934.1	*Oryza coarctata*	LC739565.1	*Oryza sativa* Japonica Group

Furthermore, Yunnan *Oryza rufipogon* showed a closer relationship with *Oryza nivara*, and both widely distributed in Asia. Yunnan *Oryza officinalis* possibly originated from and evolved with *Oryza australiensis*, and both mainly distributed in subtropical regions. The Yunnan Oryza *granulata*, *Oryza brachyantha*, *Oryza longiglumis*, and *Oryza ridleyi* clearly clustered into one clade with 100% bootstrap support, indicating that their congeners are closely related. Additionally, the genetic relationship between the Yunnan *Oryza granulata* and *Oryza brachyantha* was closer. *Oryza schlechteri* was located in the next clade of the Yunnan *Oryza granulata* with 100% bootstrap support. The three wild rice species possessed distinguish dwarf trait and tolerance to shade from other *Oryza* species. However, *Oryza brachyantha* and *Oryza schlechteri* belonged to the unclassified group of the Vaughan-Williams classification system. Notably, *Oryza coarctata* clustered with the clade of Japonica cultivated rice, distinctly separated from Indica by *Oryza rufipogon*, compared with previous reports. Furthermore, it was confirmed that the subspecies Geng(j) (Japonica) and Xian(i) (Indica) were independently domesticated. The phylogenetic tree suggests that the unclassified wild rice species *Oryza schlechteri* and *Oryza brachyantha* may have been derived from *Oryza officinalis* and Malay wild rice *Oryza ridleyi*, respectively, and ecological pressures significantly effect on the evolution of cp genome, confirming the reliability of the assembly of cp genomes of the Yunnan wild rice species *Oryza rufipogon*, *Oryza officinalis*, and *Oryza granulata* and produced a well-resolved and strongly supported classification and evolutionary relationship for Yunnan wild rice species in the genus *Oryza*.

### 3.3 Codon usage bias analysis of cp genomes of the three wild rice species and *Oryza sativa*

#### 3.3.1 Codon correlation and ENC value analysis

The CAI refers to the fitness coefficient when all codons encode a protein using the optimal codon relative to the gene. The CAI value is between 0 and 1, and a higher value means a likely stronger codon usage bias and a potential higher expression level. The ENC value, ranging from 20 to 61, is an important indicator of the degree of codon usage bias within a gene. An ENC value of 20 indicated extreme bias when only one codon was used for each amino acid, whereas an ENC value of 61 corresponded to all codons uniformly used. An ENC value of 45 was used as a criterion to distinguish the degree of codon bias; ENC < 45 indicates strong gene bias, whereas ENC > 45 indicates weak bias (Bulmer, 1987; Ikemura, 1985; Hu et al., 2024; Zhang and Feng, 2025). CodonW calculates CAI and ENC value that measure the degree of adaptation of a gene’s codon usage to optimal codon usage (**Table 4**). The GC content varied at different positions, with the first, second, and third positions in the codon showing the following trend: GC1 > GC2 > GC3; this indicated that the cp genome sequences of *Oryza* spp. are rich in A/T bases, particularly at the third position of the codons. The average ENC values in the cp genome were 48.09–49.43, which were > 45. Furthermore, all four species had the same average CAI value of 0.17. These results indicate that the overall codon bias in the cp genomes was relatively weak.

**Table 4:** Fundamental parameters of codon usage bias of cp genomes.

Species	GC1%	GC2%	GC3%	ENC	CAI
*O. rufipogon*	47.07	39.80	31.01	49.32	0.17
*O. officinalis*	47.20	39.60	30.69	49.43	0.17
*O. granulata*	47.26	39.52	30.71	48.08	0.17
*O. sativa*	46.83	39.32	30.43	49.25	0.17

ENC, effective number of codons; CAI, codon adaptation index.

**Figure 4:**
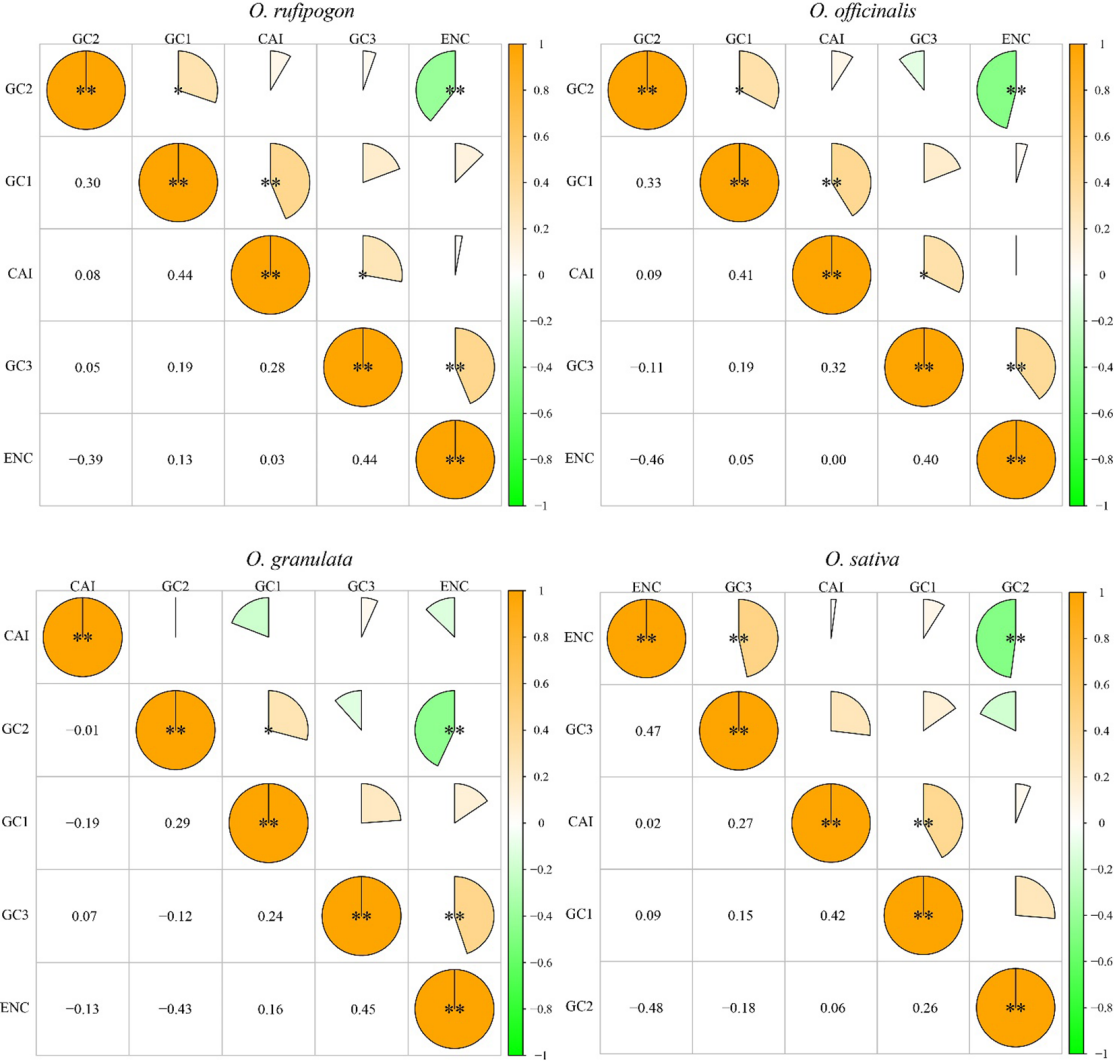
Correlation analysis of codon parameters in chloroplast genomes. A higher value and larger sectorial area indicate a more significant correlation. *indicates significant difference at 0.05 level; **indicates significant difference at 0.01 level. CAI refers to the codon adaptation index. ENC refers to an important indicator that influences codon preference. GC1, GC2, and GC3 represent the GC content of the first to the third base on the codon. The significant positive correlation between CAI and GC1 in *Oryza rufipogon*, *Oryza officinalis*, and *Oryza sativa* suggests that the changes at the first codon position were determined using the optimal codons. The significant negative correlation between ENC and GC2 in three Yunnan wild rice species and cultivated rice indicates that the GC content of the 2 codon has an impact on codon preference.

Furthermore, the correlation between the GC content, ENC, and CAI of the cp genome CDS was analyzed (**Figure 4**). The results showed a significant positive correlation between CAI and GC1 in *Oryza rufipogon*, *Oryza officinalis*, and *Oryza sativa* (p < 0.01) as well as between CAI and GC3 in *Oryza rufipogon* and *Oryza officinalis* (p < 0.05) but no significant correlation between CAI and GC1 or GC3 in *Oryza granulata* (p > 0.05). This indicates that the changes at the first and third codon positions were determined using the optimal codons due to a higher CAI value meaning a likely stronger codon usage bias in *Oryza rufipogon* and *Oryza officinalis*. In the Yunnan wild rice species, there was a significant positive correlation between GC1 and GC2 (p < 0.05), implying that the base composition of the first two codon positions was similar but significantly different from that of the third codon position. Additionally, there was a positive correlation between ENC and GC3 (p < 0.01) and a significant negative correlation between ENC and GC2 (p < 0.01). This revealed that the codon usage bias of the cp genomes in the four species was primarily determined by the bases at the second and third codon positions. The similarities and differences in the correlations among the four species indicate that the commonalities and subtle differences in codon usage bias may be important characteristics reflecting the differences in their phylogenetic relationships.

#### 3.3.2 Optimal codon analysis

High- and low-expression gene datasets were set up according to the ENC values of each CDS. The RSCU and ΔRSCU values were calculated as presented in **Table 5**. The optimal codons in four species were determined according to the ΔRSCU values. Comparative analysis revealed that *Oryza rufipogon*, *Oryza officinalis*, *Oryza granulata*, and *Oryza sativa* contained 17, 15, 16, and 15 high-frequency and highly expressed codons, respectively. Among these, the 14 codons of UUA, AUU, GUA, UCC, CCU, ACU, GCU, UAA, CAA, AAA, CGU, AGU, AGA, and GGU were the most commonly favored optimal codons by the four species. In all four species, only one codon in the cp genome tended to end at C, while the other 13 optimal codons tended to end at A/U, indicating that the codon usage bias of the cp genome of the four species tended to end at A/U, which could be important for improving gene expression.

**Table 5:** RSCU analysis of amino acids in cp genomes.

Codon	*O. rufipogon*		*O. officinalis*		*O. granulata*		*O. sativa*	
	△RSCU	RSCU	△RSCU	RSCU	△RSCU	RSCU	△RSCU	RSCU
AAA*	0.66	1.47	0.32	1.46	0.35	1.47	0.66	1.46
AAC	0.49	0.53	0.57	0.53	0.66	0.53	0.49	0.53
AAG	-0.66	0.53	-0.32	0.54	-0.35	0.53	-0.66	0.54
AAU	-0.49	1.47	-0.57	1.47	-0.66	1.47	-0.49	1.47
ACA	-0.06	1.06	0.03	1.09	0.09	1.06	-0.06	1.11
ACC	-0.21	0.78	-0.12	0.76	-0.54	0.76	-0.21	0.73
ACG	-0.76	0.45	-0.94	0.47	-0.72	0.47	-0.76	0.45
ACU*	1.03	1.71	1.03	1.68	1.17	1.71	1.03	1.70
AGA*	0.14	1.69	0.45	1.68	0.18	1.64	0.14	1.69
AGC	-0.47	0.39	-0.18	0.38	-0.03	0.39	-0.47	0.41
AGG	-0.37	0.63	-0.35	0.59	-0.35	0.64	-0.37	0.61
AGU*	0.60	1.28	0.22	1.27	0.42	1.26	0.60	1.28
AUA	-0.11	0.90	-0.30	0.91	-0.21	0.90	-0.11	0.91
AUC	-0.05	0.59	-0.05	0.59	-0.13	0.59	-0.05	0.58
AUG	0.00	1.00	0.00	1.00	0.00	1.00	0.00	1.00
AUU*	0.17	1.51	0.35	1.51	0.34	1.50	0.17	1.51
CAA*	0.19	1.51	0.16	1.52	0.30	1.52	0.19	1.51
CAC	0.01	0.52	0.04	0.55	0.16	0.52	0.01	0.51
CAG	-0.19	0.49	-0.16	0.48	-0.29	0.48	-0.19	0.49
CAU	-0.01	1.48	-0.04	1.45	-0.16	1.48	-0.01	1.49
CCA	-0.05	1.06	-0.17	1.08	-0.07	1.08	-0.05	1.06
CCC	-0.74	0.88	-0.49	0.95	-0.37	0.88	-0.74	0.90
CCG	-0.48	0.47	-0.64	0.46	-0.86	0.46	-0.48	0.46
CCU*	1.28	1.59	1.30	1.51	1.30	1.59	1.28	1.58
CGA	-0.41	1.27	-0.12	1.32	-0.02	1.32	-0.41	1.32
CGC	-0.38	0.57	-0.22	0.54	-0.31	0.56	-0.38	0.58
CGG	-0.09	0.50	-0.47	0.49	-0.22	0.46	-0.09	0.49
CGU*	1.11	1.34	0.71	1.39	0.72	1.39	1.11	1.30
CUA	-0.31	0.82	-0.07	0.82	-0.27	0.83	-0.31	0.80
CUC	-0.46	0.41	-0.48	0.42	-0.29	0.40	-0.46	0.40
CUG	-0.19	0.35	-0.36	0.35	-0.13	0.35	-0.19	0.34
CUU	-0.07	1.30	-0.09	1.29	-0.41	1.30	-0.07	1.31
GAA	-0.41	1.47	-0.27	1.48	-0.32	1.47	-0.41	1.48
GAC	-0.07	0.44	0.34	0.46	0.09	0.44	-0.07	0.42
GAG	0.41	0.53	0.27	0.52	0.32	0.53	0.41	0.52
GAU	0.07	1.56	-0.33	1.54	-0.09	1.56	0.07	1.58
GCA	-0.02	1.19	-0.11	1.19	-0.52	1.18	-0.02	1.18
GCC	-0.11	0.56	-0.14	0.56	-0.05	0.56	-0.11	0.56
GCG	-0.22	0.51	-0.26	0.48	-0.14	0.50	-0.22	0.51
GCU*	0.34	1.75	0.51	1.77	0.71	1.76	0.34	1.75
GGA	-0.47	1.53	-0.19	1.53	-0.65	1.52	-0.47	1.55
GGC	-0.17	0.43	0.07	0.43	0.20	0.44	-0.17	0.40
GGG	-0.46	0.76	-0.61	0.77	-0.48	0.77	-0.46	0.77
GGU*	1.09	1.28	0.73	1.27	0.93	1.27	1.09	1.28
GUA*	0.66	1.47	0.63	1.47	0.60	1.45	0.66	1.46
GUC	-0.29	0.45	-0.34	0.46	-0.45	0.44	-0.29	0.45
GUG	-0.04	0.53	-0.34	0.53	-0.16	0.54	-0.04	0.52
GUU	-0.34	1.55	0.05	1.54	0.01	1.57	-0.34	1.57
UAA*	1.20	1.50	0.60	1.44	0.60	1.50	1.20	1.50
UAC	0.34	0.45	0.23	0.44	0.18	0.44	0.34	0.44
UAG	-1.20	0.75	-0.60	0.84	-1.20	0.78	-1.20	0.75
UAU	-0.34	1.55	-0.23	1.56	-0.18	1.56	-0.34	1.56
UCA	-0.43	1.04	-0.94	1.03	-0.91	1.06	-0.43	1.06
UCC*	0.40	1.20	0.55	1.20	0.26	1.17	0.40	1.17
UCG	-0.18	0.52	-0.44	0.53	-0.38	0.53	-0.18	0.54
UCU	0.05	1.56	0.77	1.60	0.64	1.59	0.05	1.54
UGA	0.00	0.75	0.00	0.72	0.60	0.72	0.00	0.75
UGC	-0.47	0.51	0.29	0.53	-0.75	0.52	-0.47	0.51
UGG	0.00	1.00	0.00	1.00	0.00	1.00	0.00	1.00
UGU	0.47	1.49	-0.29	1.47	0.75	1.48	0.47	1.49
UUA*	1.36	2.01	0.73	2.00	1.21	2.01	1.36	2.05
UUC	0.16	0.70	0.21	0.70	0.43	0.70	0.16	0.69
UUG	-0.34	1.11	0.28	1.13	-0.11	1.12	-0.34	1.10
UUU	-0.16	1.30	-0.21	1.30	-0.43	1.30	-0.16	1.31

*, optimal codon of four rice species.

RSCU, relative synonymous codon usage.

#### 3.3.3 ENC-plot analysis of the degree of codon usage bias

**Figure 5:**
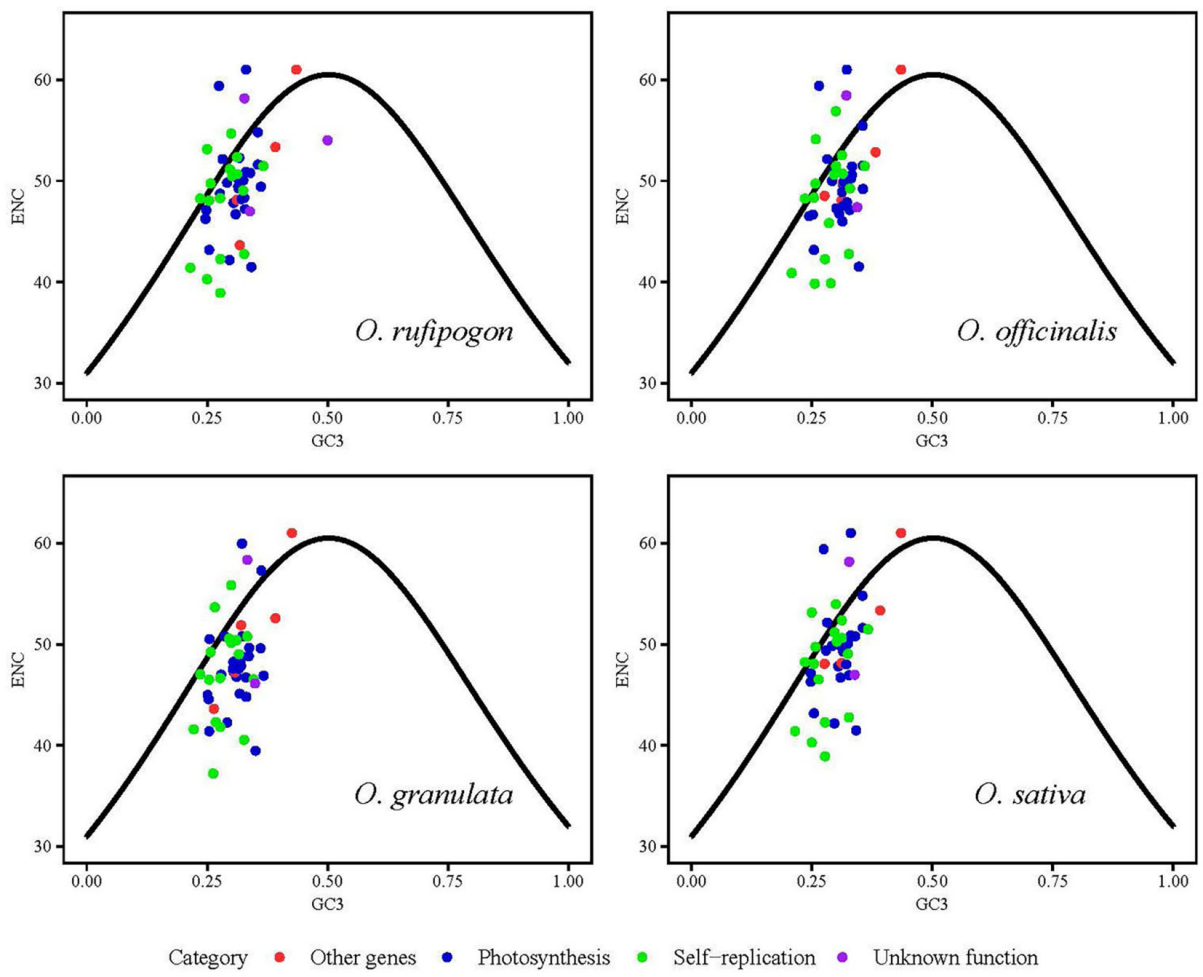
Analysis of the effect of nucleotide composition on codon usage bias using effective number of codons (ENC)-plot. GC3 is defined as the GC content of the synonymous codon’s third position. ENCexp and ENCobs indicate the expected and actual observed values of ENC, respectively. If a data point is significantly distant from the standard curve, this indicates that the codon usage bias of chloroplast coding sequences is predominantly influenced by natural selection.

**Figure 6:**
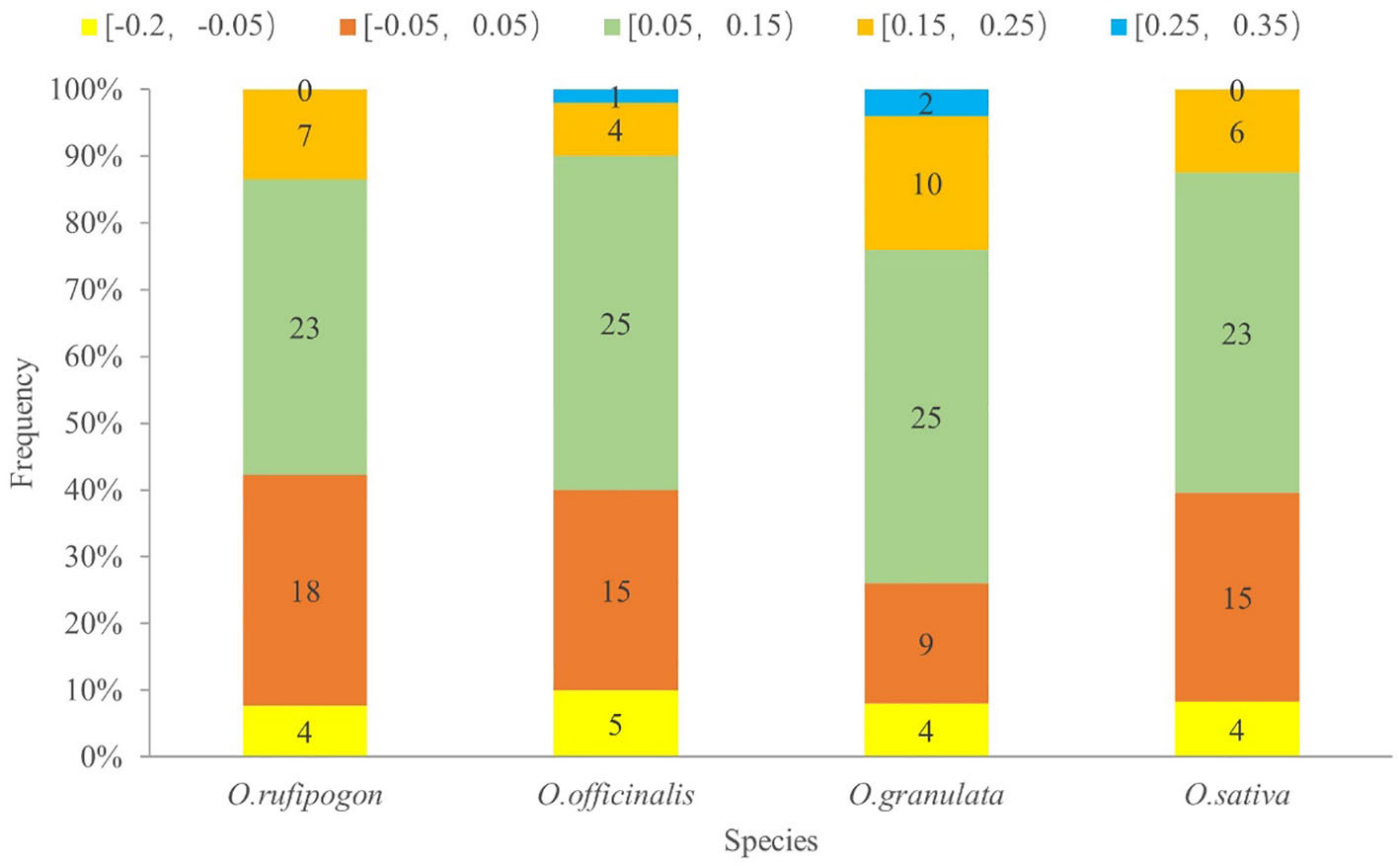
Frequency distribution of the ENC ratio. The horizontal axis is the value of the ENC ratio [(ENCexp - ENCobs)/ENCexp]; the vertical axis indicates the number of genes of cp genomes of four species. Different colors in a column represent distributed ranges. If codon usage bias is predominantly influenced by mutational pressure, then the ENC ratio of most genes should fall within the range of −0.05 to 0.05.

To analyze the effect of the GC content of the third base of the codon position on codon usage bias, ENC values were calculated and are showed in **Figure 5**. This formula described in section 2.4.2 was used as the standard curve, and the vertical distance between the point and the standard curve was used as the evaluation index. If the point was located on the standard curve, it indicated that codon usage bias was mainly affected by gene mutations; otherwise, it was primarily affected by natural selection. **Figure 5** shows that most of the genes were located below the standard curve, and the photosynthesis- and self-replication-related genes were relatively far away from the vertical distance of the standard curve, indicating that the codon usage bias of this type of gene was mainly affected by natural selection. To investigate a more accurate relationship between expected ENC ​​(ENCexp) and observed ENC values (ENCobs), the ENC ratio and their distribution were presented in **Supplementary Table 2** and **Figure 6**. The calculation of ENC ratio using the formula of ENCexp - ENCobs)/ENCexp revealed that *Oryza rufipogon*, *Oryza officinalis*, *Oryza granulata*, and *Oryza sativa* had 18 (35%), 15 (30%), 9 (18%), and 15 (31%) genes, respectively, with ENC ratios distributed between −0.05 to 0.05, and ENCobs was relatively close to the ENCexp value. Most of genes with ENC ratios outside the range of −0.05 to 0.05 exhibited a significant deviation between ENCobs and ENCexp, which indicated that their codon usage bias was more influenced by natural selection. These results indicates that the codons in the cp genomes of *Oryza rufipogon*, *Oryza officinalis*, and *Oryza sativa* were mutually determined by natural selection and mutation pressure, with natural selection as the dominant force, whereas 82% of the codons in *Oryza granulata* were affected by natural selection.

#### 3.3.4 PR2-plot analysis of factors affecting codon usage bias

**Figure 7:**
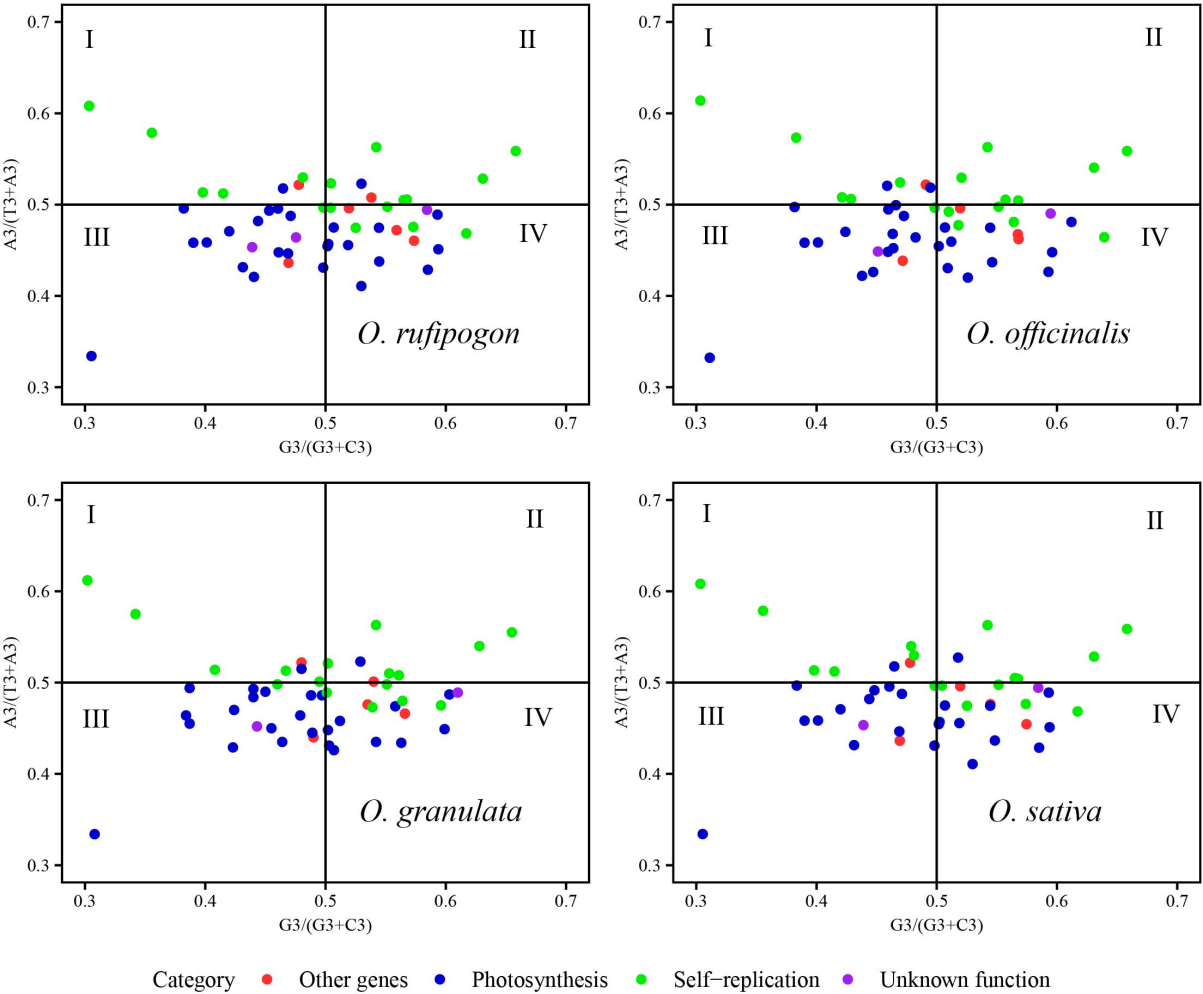
Analysis of factors influencing codon usage bias using a PR2-plot. The GC bias is plotted on the x-axis, while the AT bias is plotted on the y-axis. The A3, T3, G3, and C3 represent the frequency of A/T/G/C at the third position of synonymous codons.

The usage patterns of A/T and G/C at the third codon position in the cp genomes of the four species were analyzed to explore the multiple factors affecting codon usage bias using a PR2-plot with RStudio described in section 2.4.1 and 2.4.2 (**Figure 7**). In the PR2-plot, the central point represents A = T and G = C, and each point represents a coding gene. If the coding gene fell on or near the central point, the main factor affecting codon usage bias would be mutations; otherwise, it would be influenced by natural selection and mutations. In this study, most coding genes from *Oryza rufipogon*, *Oryza officinalis*, and *Oryza sativa* were distributed in the quadrant III and IV of the central point and there were some more genes in the quadrant IV than quadrant III, indicating that the frequency of code usage at the third code position was A < T and C < G. However, *Oryza granulata* coding genes were mainly scattered at the quadrant III and IV of the central point and there were some more genes in the quadrant III than quadrant IV, implying that the frequency of code usage at the third code position was A < T and C > G. Compared to the unknown function genes and other genes, the coding genes related to self-replication and the photosynthetic system were more scattered to the central point. Therefore, the codon usage bias of the cp genomes of the four species was simultaneously affected by natural selection and mutations; however, natural selection was the major driving force.

#### 3.3.5 Analysis of factors influencing codon usage bias using a neutrality plot

**Figure 8:**
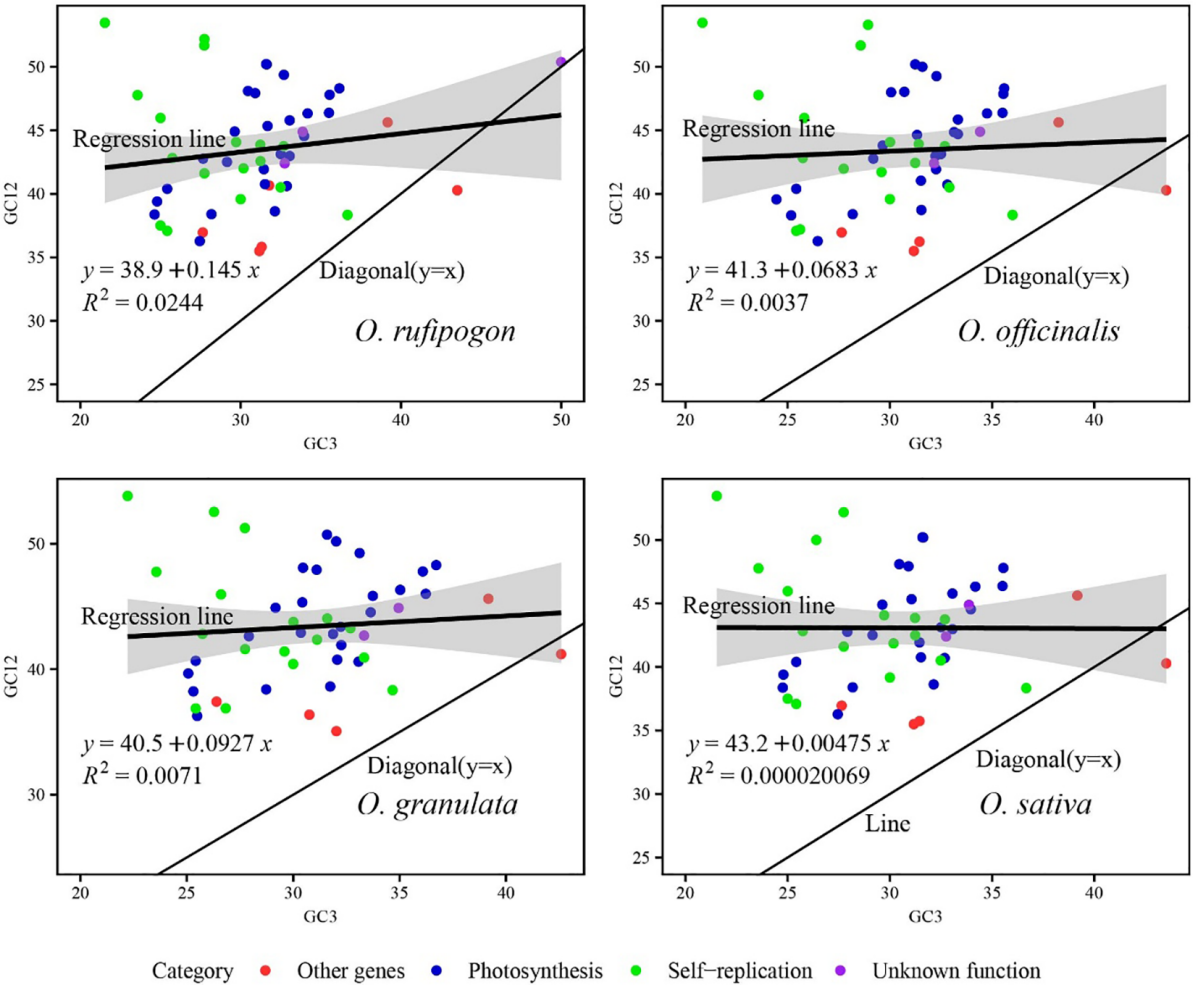
Analysis of factors influencing codon usage bias using a neutrality plot. The black line in the plot displays the correlation trend, with the corresponding equation showed at the left bottom of the graph. The value GC12 represents the average GC content of the codon’s first two bases. R stands for Pearson’s correlation coefficient. In the plot for *O. rufipogon*, the correlation line is straight and have larger slope of 0.145 corresponding to a stronger correlation between GC12 and GC3 compared to other two Yunnan wild rice species and cultivated rice. Whereas, the correlation line is straight and parallel to the x-axis with smaller slope of 0.00475 corresponding to a weaker correlation in the plot for *O. sativa*. Generally, the weak correlation between GC12 and GC3 in the four species suggests the codon usage bias is mainly affected by natural selection.

A neutrality plot was constructed to assess the extent of mutation pressure against natural selection on the codon usage bias of the cp genome. A significant correlation between GC12 and GC3 indicated similar base composition at the third codon position and mutational force as the influencing factor of codon usage bias. If there is no significant correlation between them, as the slope gradually decreased or diminished to zero, the effect of natural selection on the codon usage bias progressively strengthened. As showed in **Figure 8**, the genes were predominantly distributed above the diagonal (y=x) line, with only one other gene locating below the diagonal and one unknown function gene lying along the diagonal. Compared with other three function genes, the self-replication-related genes were more scattered to the regression line (dark thick black lines in the figures with shaded area as confidence interval), indicating that the codon usage bias of these genes was mainly affected by natural selection. The slope of the regression line fitted with GC12 and GC3 ranged from 0.00475 to 0.145, with R^2^ values of 0.00002–0.0244, which indicated significant differences in base content at different positions and a weak correlation between GC12 and GC3 (p>0.05). It was inferred that the mutation patterns of the first and second bases were different from those of the third base, and codon usage bias was more affected by natural selection than by mutations in the cp genomes of the four species.

## 4 Discussion

The cp genomes of higher plants typically possess a conserved circular tetrad structure with a high degree of sequence similarity and a relatively high divergence of intergenic spacers (Li et al., 2023a; Yuan et al., 2023; Zhang et al., 2024). In this study, we sequenced, assembled, and annotated the cp genomes of the Yunnan wild rice species. The genome sizes of *Oryza rufipogon*, *Oryza officinalis*, and *Oryza granulata* were 134,824, 134,587, and 135,937 bp, respectively, with a GC content of 39.0%. Among *Triticum aestivum* (Middleton et al., 2014), *Zea mays* (Wang et al., 2024b), *Sorghum bicolor* (Saski et al., 2007), *Secale cereale* (Middleton et al., 2014), *Oryza rufipogon*, *Oryza officinalis*, and *Oryza granulata*, it was showed that the size of the cp genome of the three Yunnan wild rice species was between the largest cp genome of *Sorghum bicolor* with 140,752 bp and the smallest of *Secale cereale* with 114,843 bp, with GC content ranging from 37% (*Secale cereale*) to 39.0% (Yunan wild rice species). This indicates that cp genome size and composition among gramineous crops were conservative, with few differences, despite significant variation in their whole-genome size across species. Additionally, the cp genomes of *Oryza meridionalis* (KM103373) and *Oryza officinalis* (NC_027463) were 134,556 bp and 134,911 bp, respectively, which were smaller than those of *Oryza granulata* but consistent with those of *Oryza rufipogon* and *Oryza officinalis*. Upon scrutinizing the size difference between the cp genome of the Yunnan *Oryza granulata* and the other two wild rice varieties, we found that it mainly existed in the LSC region. The LSC region of the Yunnan *Oryza granulata* was 81,837 bp, which was approximately 2,000 bp larger than that of *Oryza rufipogon* (80,636 bp) and *Oryza officinalis* (LSC 80,852 bp). The LSC sizes were highly similar to those reported in previous studies, which might be why a larger LSC led to a longer cp genome of *Oryza granulata* compared with the other two wild rice species (Wambugu et al., 2015; Song et al., 2017; Gao et al., 2019). Reportedly, the GC content is crucial for gene stability (Hao et al., 2022). In the tetrad structure of the cp genome, the GC content in the IR region was higher than that in the LSC and SSC regions, further confirming that the GC content in the IR region determines the conservation of the cp genome.

In higher plants, cp physiological functions are closely related to leaf development and function, and cp development and gene expression are primarily determined by nuclear genes. Therefore, exploring nuclear genes located in chloroplasts, particularly those with regulatory functions, is vital for chloroplast-related molecular biology research (Kaiser et al., 2019). At the Third International Barcode of Life Conference, the chloroplast barcoding regions of MaturaseK (*MatK*) and ribulose-1,5-bisphosphate carboxylase/oxygenase large subunit (*rbcL*) genes were identified as the core standard DNA barcode loci for plants. The intergenic spacer region *trnH*–*psbA* and the nuclear gene region internal transcribed spacer were used as supplementary codes for the standard DNA barcode loci. Our study identified four main groups within the cp genome: photosynthetic system genes, self-replication-related genes, other genes, and unknown functional genes. Most genes were related to the photosynthetic system, including all genes and regions of the core and the supplementary code of plant standard barcodes. As the cp genome is self-replicating, except for photosynthesis-related genes, the second-largest genes identified were self-replication-related genes. That is, the cp genome cannot encode all of the proteins required for photosynthesis, necessitating their combination with the proteins encoded by the nuclear genome to form composites with photosynthetic activity to exert their functions. Many self-replication-related genes are present in the cp genome. Comparing the cp genome genes of the three Yunnan wild rice species and cultivated rice revealed that only four of the 133 genes showed the existence or absence of differences. The very few different genes among the four species further confirmed that the cp genome was relative conserved in terms of gene content in the three Yunnan wild rice species and cultivated rice. Notably, the three Yunnan wild rice species possessed *ycf68* whereas the cultivated *Oryza sativa* rice did not, suggesting that *ycf68* disappeared during rice domestication. However, the timing and reasons for this require further investigation.

Vaughan (1994) developed a classification system that combined pedigree analysis with chromosome grouping, which is the most widely accepted system for the genus *Oryza*. It divided 22 species of *Oryza* into five groups: *Oryza officinalis*, *Oryza granulata*, *Oryza sativa*, *Oryza ridleyi*, and an unclassified group, which includes *Oryza brachyantha* and *Oryza schlechteri*. In this study, the evolutionary relationships of 25 species of *Oryza* based on the phylogenetic tree analysis of cp genomes were consistent with the five grouping categories in the Vaughan system. Among them, *Oryza rufipogon*, *Oryza officinalis*, and *Oryza granulata* were clustered in the corresponding branches with 100% bootstrap support (Zhu et al., 2014). *Oryza rufipogon* and *Oryza nivara* were the two wild species most closely related to *Oryza sativa*, which is similar to previous results (Zhou et al., 2008; Zhu et al., 2014). Notably, *Oryza rufipogon* clustered with the clade of cultivated Japonica rice, distinct from Indica. These results further confirmed that the subspecies Geng (j) (Japonica) and Xian (i) (Indica) were independently domesticated. Furthermore, *Oryza coarctata* was reported as the third closest wild species to *Oryza sativa*. In Yunnan, *Oryza officinalis* might evolve from *Oryza australiensis*, consistent with previous studies (Zhu and Ge, 2005; Zou et al., 2008; Gao et al., 2019). The genetic relationship between Yunnan *Oryza granulata* and *Oryza brachyantha* according to the cp genome, was the closest, with 100% bootstrap support. This finding is similar to that reported by Gao et al. (2019). Additionally, we initially found that *Oryza schlechteri* might be differentiated from *Oryza granulata*, which is present in the next branch of *Oryza granulata* with 100% bootstrap support. The unclassified wild rice group may be classified as *Oryza ridleyi* or the *Oryza granulata* groups, which was further verified by Asaf et al. (2017). Therefore, the evolutionary relationships among Yunnan wild rice species in the genus *Oryza* can be clearly interpreted based on phylogenetic tree construction using cp genome sequencing. Insights from complete cp genome sequences have enhanced our understanding of the biology and diversity of wild rice species, and cp genomes have made significant contributions to resolving the evolutionary relationships within phylogenetic clades. It is highly recommended to use cp genome sequencing for evolutionary relationships in plants because of the conservation and small size of the cp genome, which reduces the sequencing cost.

Analysis of codon usage bias in the cp genomes of the three Yunnan wild rice species and *Oryza sativa* showed that the average ENC values of the CDS were > 45, indicating weak codon usage bias of the genus *Oryza*. Strong codon usage bias may impede the response of the genome to rapid and intensifying climate change and ecosystems. The weak codon usage bias of the genus *Oryza* is possibly caused by its evolutionary adaptation (Chi et al., 2020). The optimal codons UUA, AUU, GUA, UCC, CCU, ACU, GCU, UAA, CAA, AAA, CGU, AGU, AGA, and GGU were further identified in this study, most of which ended with A/U, and the results align with those of *Elaeagnus* (Li et al., 2023b), *Gynostemma* (Zhang et al., 2021) and *Rutaceae* (Shen et al., 2024), indicating that there were some similarities in optimal codons among different species. Moreover, the similarities facilitate the optimization of target gene codons, improving the efficiency of heterologous gene expression vectors, thereby accelerating the target gene to match the structural features of the host genes and significantly enhancing the likelihood of high-level protein expression (Watts et al., 2021). Correlation analysis showed that GC2 and GC3 significantly influenced the codon usage bias in the Yunnan wild rice and *Oryza sativa* chloroplast genomes. ENC-plot, PR2-plot, and neutral plot analyses showed that the codon usage bias of Yunnan wild rice and *Oryza sativa* was affected by natural selection and mutation pressure, with natural selection significantly affecting the codon usage bias of *Oryza granulata*. Furthermore, the three analyses showed that the codon usage bias of most photosynthesis and self-replication genes was influenced by natural selection. This may explain the remarkable differences in photosynthetic characteristics: *Oryza rufipogon* and *Oryza officinalis*, with codon usage bias being mutually affected by natural selection and genetic mutation, preferred humid swamps with higher photosynthetic efficiency, while shade-exposed and upland wild rice species *Oryza granulata*, with codon usage bias being mainly influenced by natural selection, optimized photosynthesis to adapt to shaded environments.

In the present study, the cp genomes of three Yunnan wild rice species were sequenced, assembled, and annotated to better understand their genomic composition and genetic information. Additionally, codon usage bias was compared between Yunnan wild rice and *Oryza sativa* rice, providing a theoretical basis and guidance for further investigation of the transcription and translation of exogenous genes in genetic engineering, gene function, and species evolution. Future research should integrate nuclear genes, molecular markers, and specific gene fragments to verify evolutionary relationships within the genus *Oryza* based on the cp genome. Since cp functional genes are pleiotropic and may regulate multiple traits (Cejudo et al., 2021; Gao et al., 2023), nuclear genome, proteomic, and biochemical approaches are needed to synergistically characterize the molecular mechanisms of cp gene expression in wild rice species.

## Data Availability

The data reported in this paper have been deposited in the GenBase in National Genomics Data Center, Beijing Institute of Genomics, Chinese Academy of Sciences/China National Center for Bioinformation, under accession number C_AA107112.1 to C_AA107114.1 that is publicly accessible at https://ngdc.cncb.ac.cn/genbase.
