# Supplementary material for: Characterization of the complete chloroplast genome and comparative analysis of the phylogeny and codon usage bias of three Yunnan wild rice species
Source: Front Plant Sci. 2025 Jul 2;16:1555104. doi: 10.3389/fpls.2025.1555104 (PMC12264640; doi:10.3389/fpls.2025.1555104)
Supplement: Supplementary file 1 [file DataSheet1.zip › Supplementary Table 1.docx]

Supplementary Table 1. Data quality information of chloroplast genome of three Yunnan wild rice species

| Species | Raw data | Clean data | Q20% | Q30% | GC% |
| --- | --- | --- | --- | --- | --- |
| *O. rufipogon* | 1.87Gb | 1.73Gb | 95.71 | 91.22 | 44.32 |
| *O. officinalis* | 1.41Gb | 1.29Gb | 97.16 | 92.67 | 44.22 |
| *O. granulata* | 1.9Gb | 1.8Gb | 97.43 | 93.67 | 56.14 |
